# Lipid Mediators and Cytokines/Chemokines Display Differential Profiles in Severe versus Mild/Moderate COVID-19 Patients

**DOI:** 10.3390/ijms241713054

**Published:** 2023-08-22

**Authors:** Resmi Ravindran, Ellen O’Connor, Ajay Gupta, Paul A. Luciw, Aleena I. Khan, Nasrin Dorreh, Kate Chiang, Aamer Ikram, Srinivasa Reddy

**Affiliations:** 1 Department of Pathology and Laboratory Medicine, University of California, Davis, CA 95817, USA; paluciw@ucdavis.edu; 2 Division of Cardiology, Department of Medicine, David Geffen School of Medicine, University of California, Los Angeles, CA 90095, USA; eioconnor@ucla.edu (E.O.); ndorreh@mednet.ucla.edu (N.D.); katecc789@outlook.com (K.C.); 3 Division of Nephrology, Hypertension and Kidney Transplantation, Department of Medicine, University of California Irvine (UCI) School of Medicine, Irvine, CA 92868, USA; ajayg1@hs.uci.edu; 4 Department of Population and Public Health, Keek School of Medicine, University of Southern California, Los Angeles, CA 90089, USA; aikhan@usc.edu; 5 National Institutes of Health, Islamabad 45500, Pakistan; maahin1@yahoo.com

**Keywords:** COVID-19, cytokines, lipid mediators

## Abstract

Host immune responses play a key role in COVID-19 pathogenesis. The underlying phenomena are orchestrated by signaling molecules such as cytokines/chemokines and lipid mediators. These immune molecules, including anti-SARS-CoV-2 antibodies, interact with immune cells and regulate host responses, contributing to inflammation that drives the disease. We investigated 48 plasma cytokines/chemokines, 21 lipid mediators, and anti-S protein (RBD) antibodies in COVID-19 patients (n = 56) and non-COVID-19 respiratory disease controls (n = 49), to identify immune-biomarker profiles. Cytokines/chemokines (IL-6, CXCL-10 (IP-10), HGF, MIG, MCP-1, and G-CSF) and lipid mediators (TxB2, 11-HETE, 9-HODE, 13-HODE, 5-HETE, 12-HETE, 15-HETE, 14S-HDHA, 17S-HDHA, and 5-oxo ETE) were significantly elevated in COVID-19 patients compared to controls. In patients exhibiting severe disease, pro-inflammatory cytokines/chemokines (IL-6, CXCL-10, and HGF) and anti-SARS-CoV-2 antibodies were significantly elevated. In contrast, lipid mediators involved in the reduction/resolution of inflammation, in particular, 5-HETE, 11-HETE, and 5-oxoETE, were significantly elevated in mild/moderate disease. Taken together, these immune-biomarker profiles provide insight into immune responses related to COVID-19 pathogenesis. Importantly, our findings suggest that elevation in plasma concentrations of IL-6, CXCL-10, HGF, and anti-SARS-CoV-2 antibodies can predict severe disease, whereas elevation in lipid mediators peaks early (compared to cytokines) and includes induction of mechanisms leading to reduction of inflammation, associated complications, and maintenance of homeostasis.

## 1. Introduction

Severe acute respiratory syndrome coronavirus 2 (SARS-CoV-2) caused hundreds of millions of confirmed cases of COVID-19, leading to approximately seven million deaths worldwide [1]. Infection with SARS-CoV-2 leads to varied clinical outcomes ranging from asymptomatic infection to mild/moderate and severe disease including acute respiratory distress syndrome (ARDS), vascular and neurological complications, and death [2,3].

As in the case of infection by pathogens in general, the outcome of virus infection is influenced by cytokines and other immunomodulating agents involved in autocrine, paracrine, and endocrine cell-signaling pathways [4]. These factors can be pro-inflammatory or anti-inflammatory and are grouped into classes such as interleukins, lymphokines, chemokines, and cell signaling molecules. Cytokines are secreted by specific immune cells and mediate interactions between cells to produce a regulated immune response toward a variety of infectious and non-infectious stimuli [5]. Correspondingly, cytokines play a crucial role in cell-regulating biological activities including effects on cell signal transduction and activation, differentiation, proliferation, and death [4,5]. Cytokines are primarily secreted by blood cells, epithelial cells, and lymphoid tissue upon induction by viral or bacterial infection [6]. Interferons (IFNs) and interleukins (IL) are specific cytokines produced by lymphocytes and epithelial cells in response to respiratory viral infections such as common coronaviruses and influenza viruses [7]. C-C motif ligand (CCL) and C-X-C motif ligand (CXCL) chemokines also serve as immunomodulators in the immune response against respiratory viruses.

The pivotal pathogenic roles of the aberrant cytokines and chemokines responses, known as a “cytokine storm”, in COVID-19 patients have drawn much attention [8]. Previous studies reported significantly higher serum levels of IL-6, IFN-α, CCL5, CXCL-8, and CXCL-10 in patients with severe disease caused by SARS-CoV or MERS-CoV compared to individuals with milder disease [9]. Several studies have established that in severe COVID-19 cases, the release of pro-inflammatory cytokines provokes the cytokine storm [10,11,12]. Our previous study in a small set of COVID-19 patients revealed that CXCL-10 levels in severe cases were consistently two-fold higher than in mild/moderate disease [13].

In addition to cytokines, lipid mediators also play a role in inflammatory processes. Eicosanoids are signaling lipids derived from polyunsaturated fatty acids including arachidonic acid (AA) and linoleic acid (LA) by cyclooxygenase (COX), lipoxygenase (LOX), and cytochrome P450 (CYP) enzymes. Lipid mediators have been known to elicit many signs of inflammation such as heat, swelling, redness, pain, and loss of function [14]. Eicosanoids include prostaglandins (PG), thromboxanes (TX), leukotrienes (LT), and lipoxins (LX) [15]. Lipid mediators also play a role in the recruitment of immune cells, cell proliferation, and migration, cytokine production, and vasoconstriction in diseases such as arthritis, atherosclerosis, cancer, dermatitis, asthma, allergic rhinitis, aortic aneurysm, ischemia/stroke, inflammatory bowel disease, and pulmonary arterial hypertension [15,16,17,18]. Additional lipid mediators including lipoxins, resolvins, protectins, and maresins help with host defense, pain, organ protection, and tissue remodeling via anti-inflammatory and pro-resolving mechanisms [19].

Lipidomic analysis of intubated COVID-19 patients showed high levels of eicosanoids in the lungs, implicating a “lipid mediator storm” in patients with severe disease (20). Bronchoalveolar lavage of control and COVID-19 patients revealed increases in thromboxane B_2_ (TXB_2_), prostaglandin D_2_ (PGD_2_), PGE_2_, PGF_2α_, leukotriene B_4_ (LTB_4_), (LTE_4_), 12-hydroxyeicosatetraenoic acid (12-HETE), 15-HETE, 5-HETE, lipoxin A4 (LXA_4_), 14-hydroxy docosahexaenoic acid (14-HDHA), 17-HDHA, resolving D1 (RvD1), RvD2, RvD4, and RvD5 [20]. Similarly, plasma analysis of COVID-19 patients showed an increase in the pro-inflammatory leukotrienes, thromboxanes, HETEs, and prostaglandins, as well as increases in the specialized pro-resolving mediators (SPMs), including 17-HDHA, RvD4, and LXA_4_, implicating a role for an eicosanoid storm, as well as a cytokine storm [21,22,23].

Early stratification of the disease on the basis of cytokine/chemokine and lipid mediator profiles may provide useful prognostic biomarkers, leading to appropriate treatment and disease management in a timely manner. Additionally, understanding such biomarker profiles may lead to the development of novel intervention strategies that target cytokine/chemokine and lipid mediator storms. In this study, plasma samples from patients with severe and mild to moderate COVID-19 were profiled for cytokines/chemokines, anti-SARS-CoV-2 antibodies, and lipidomic changes compared to non-COVID-19 respiratory disease controls. Targeted lipid mediators were compared to cytokines to identify significant differences between mild/moderate and severe COVID-19 patients. Our results suggest that concentrations of several lipid mediators and cytokines/chemokines are significantly higher in COVID-19 patients compared to disease controls. Additional analyses showed an inverse relationship in severe and mild/moderate COVID-19 between cytokines/chemokines, anti-SARS-CoV-2 antibodies, and lipidomic changes. These findings may have important implications for the identification and treatment of hyperinflammation in these patients and the development of new therapeutic targets.

## 2. Results

### 2.1. Cytokine/Chemokine and Lipid Mediator Profiles

**Figure 1:**
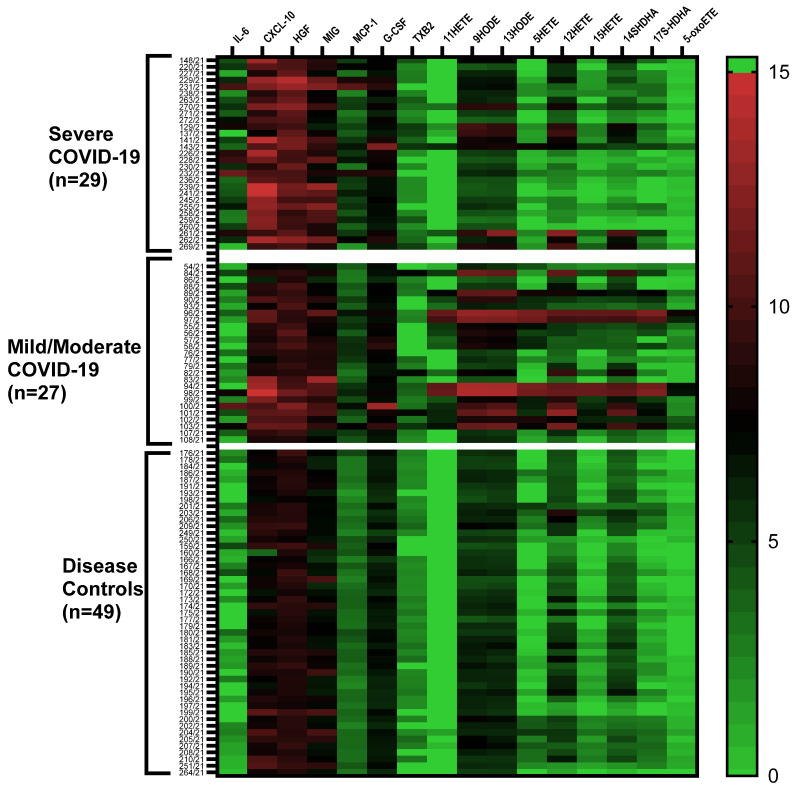
Heatmap depicting cytokines and inflammatory lipids in COVID-19 patients (n = 56; severe, n = 29; mild/moderate, n = 27) and disease controls (n = 49). Plasma concentrations of lipid mediators and cytokine/chemokines are shown as ng/mL and pg/mL, respectively. Values are log2(n + 1) transformed. Each row corresponds to one sample and columns correspond to cytokines or lipid mediators. The color intensity scale represents the log-transformed concentrations ranging from the highest (15; red) to the lowest (0; green).

Overall, profiles of cytokines/chemokines and lipid mediators in COVID-19 patients (n = 56) and disease controls (n = 49) are depicted in a heatmap (Figure 1). In general, levels of several cytokines/chemokines and lipid mediators were found to be higher in COVID-19 patients compared to disease controls (Figure 1).

**Figure 2:**
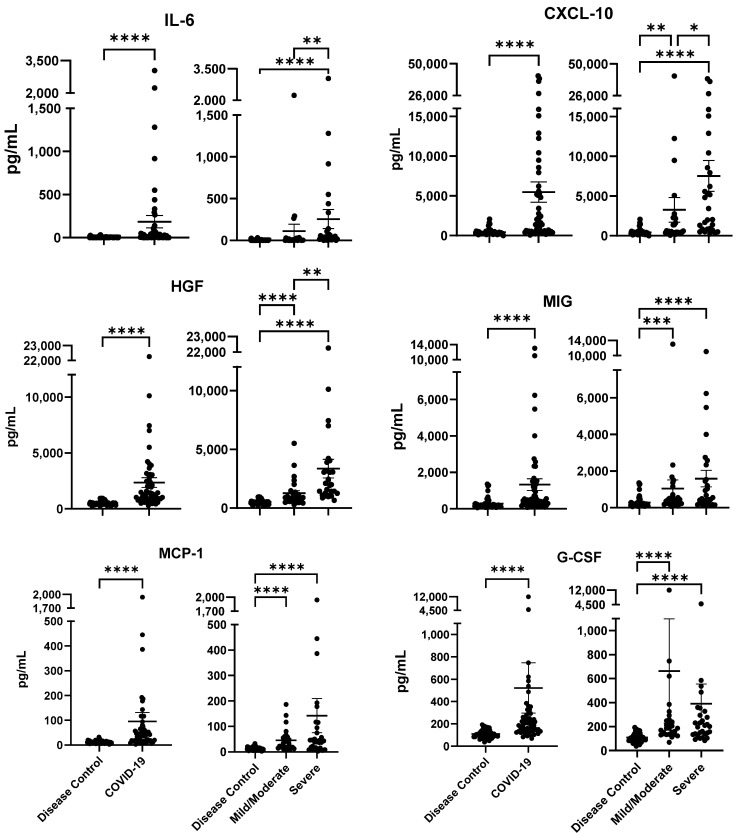
Selected cytokine/chemokines in disease controls (n = 49) vs. COVID-19 patients (n = 56; severe, n = 29; mild/moderate, n = 27). Statistical significance was tested by Mann–Whitney and Kruskal–Wallis analyses. Mean (pg/mL) ± standard error of the mean (SEM) is shown. *p*-values between the groups are as follows: * *p* < 0.05, ** *p* < 0.01, *** *p* < 0.001, **** *p* < 0.0001.

Among 48 cytokines/chemokines, evaluated by multiplex analysis in patient plasma, 6 (IL-6, CXCL-10, HGF, MIG, MCP-1, and G-CSF; Supplementary Table S2) were identified as highly significant in COVID-19 patients compared to non-COVID-19 respiratory disease controls (*p*-value < 0.0001) (Figure 2).

**Figure 3:**
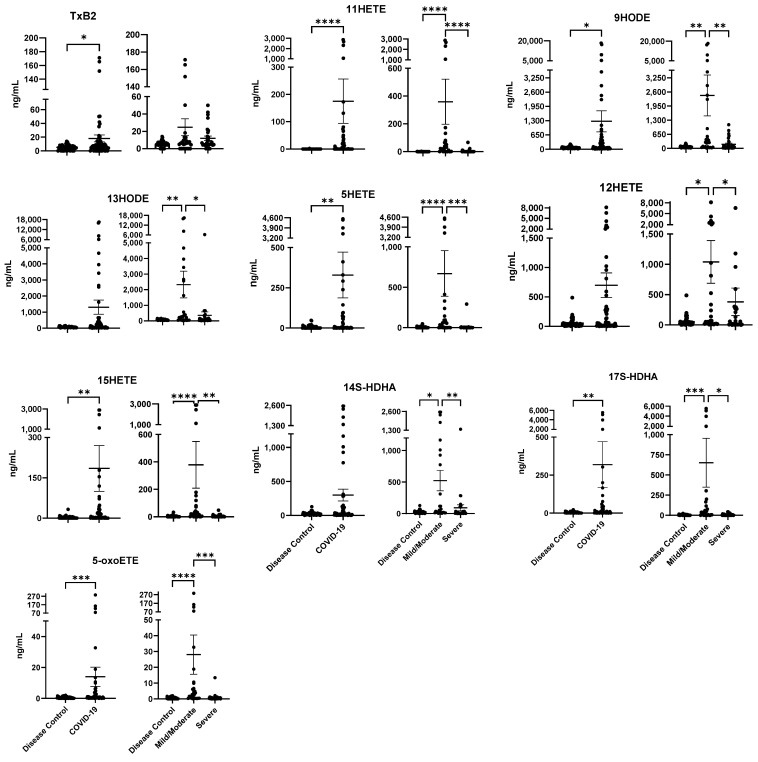
Selected plasma lipid mediators in non-COVID-19 respiratory disease controls (n = 49) vs. COVID-19 patients (n = 56; severe, n = 29; mild/moderate, n = 27). Statistical significance was tested by Mann–Whitney and Kruskal–Wallis analyses. Mean (ng/mL) ± standard error of the mean (SEM) is shown. *p*-values between the groups are as follows: * *p* < 0.05, ** *p* < 0.01, *** *p* < 0.001, **** *p* < 0.0001.

Bioactive lipid analysis, using Triple Quad 5500 (Sciex), revealed profiles of 21 distinct lipids, degradation products, and pathway markers (Supplementary Table S3). Further quantitative analysis was performed using electrospray ionization LS/MS/MS, measuring plasma concentrations of classes of pro-inflammatory, anti-inflammatory, and specialized pro-resolving lipid mediators from arachidonic acid, linoleic acid, eicosapentaenoic acid (EPA), and docosahexaenoic acid (DHA). These studies showed that profiles of eleven lipid mediators (TxB2, 11-HETE, 9-HODE, 13-HODE, 5-HETE, 12-HETE, 15-HETE, 14S-HDHA, 17S-HDHA, and 5-oxo ETE) were significantly elevated in COVID-19 patients compared to non-COVID-19 respiratory disease controls (*p*-value range: <0.001 and <0.05) (Figure 3; see left panels). In these profiles, TXB_2_ and 11HETE are pro-inflammatory lipid mediators derived from the COX pathway, while several others are derived from the LOX pathway (9HODE, 5HETE, 11HETE, 15HETE, and 5-oxo-ETE) (Figure 3; see left panels). LOX-derived anti-inflammatory downstream lipid mediators 14S-HDHA and 17S-HDHA were also significantly increased in COVID-19 patients (Figure 3; see left panels).

### 2.2. Cytokine/Chemokine Profiles Associated with COVID-19 Severity

Three cytokines/chemokines, IL-6, CXCL-10, and HGF, were found to be highly elevated in plasma samples of severe COVID-19 patients compared to mild/moderate patients (Figure 2; see right panels). The mean value for IL-6 was more than nine-fold higher in severe compared to mild/moderate patients (38 pg/mL and 4 pg/mL, respectively) (*p*-value < 0.01). The other two cytokines/chemokines were both more than two-fold higher in severe compared to mild/moderate disease, as follows: CXCL-10: 7528 pg/mL and 3267 pg/mL, respectively (*p*-value < 0.05); HGF: 2000 pg/mL and 895 pg/mL, respectively (*p*-value 0.01). The results suggest that plasma elevation of these three cytokines/chemokines may lead to severe disease by enhancing inflammatory responses.

### 2.3. Lipid Mediator Profiles Related to COVID-19 Severity

In general, all lipid mediators profiled in this study showed higher plasma concentrations in mild/moderate versus severe COVID-19 patients (*p*-value range: <0.001 and <0.05) (Figure 3; see right panels). TXB2, though not significant between mild/moderate and severe COVID-19 patients, was found in higher plasma concentrations in the former group. However, compared to non-COVID-19 respiratory disease controls, TXB2 was significantly elevated in COVID-19 patients (*p*-value < 0.05) (Figure 3). Our results demonstrate that pro-inflammatory lipid mediators of the cyclooxygenase pathway (TxB2 and 11-HETE) and the lipoxygenase pathway (9-HODE, 13-HODE, 5-HETE, 12-HETE, 15-HETE, 14-SDHA, 17-SDHA, and 5-oxo ETE,) were significantly elevated in COVID-19 patients (Figure 3; left panels of each lipid). Our observations were in agreement with previous reports [20]. However, when we analyzed our data based on the severity of the disease, we observed that all of the lipid mediators noted above were significantly elevated in the mild/moderate patient samples when compared to control (disease controls) samples or patients with severe disease (Figure 3; right panels of each lipid). Moreover, none of the resolving lipids were detected in our lipid mediator analysis.

### 2.4. Multiplex Antibody Profiles in COVID-19 Patients and Disease Controls

**Figure 4:**
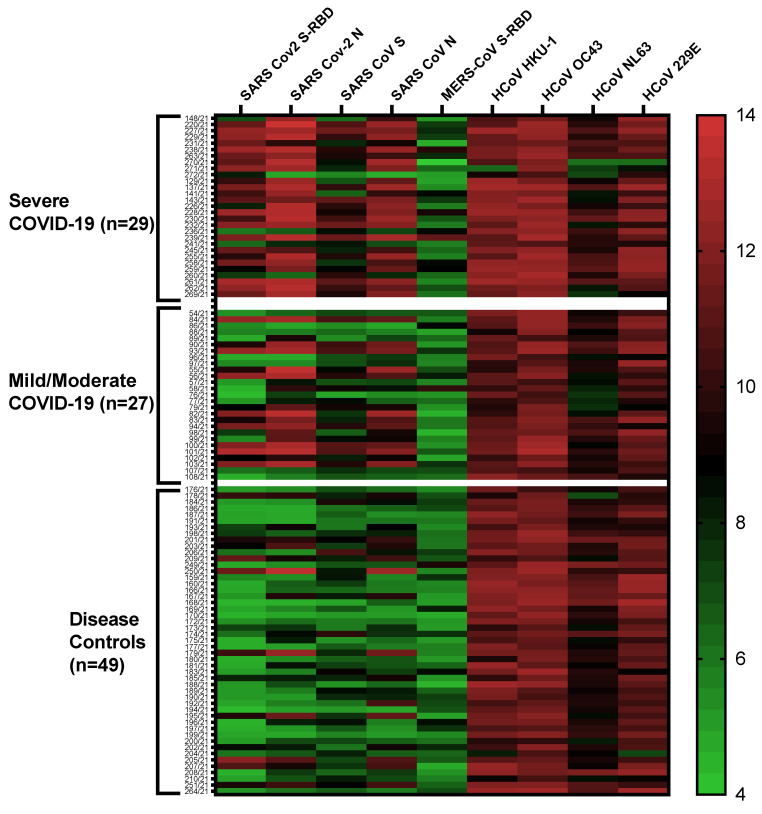
Heatmap depicting the overall antibody responses detected by multiplex microbead panel against members of the coronavirus family (SARS-CoV-2, SARS-CoV, MERS-CoV, and 4 common coronaviruses (229E, NL63, OC43, and HKU1)) in COVID-19 patients and non-COVID-19 respiratory disease controls. Each row corresponds to one sample and columns correspond to CoV antigens in the multiplex assay. The color intensity scale represents the log2 transformed MFI values ranging from the highest (14; red) to no antibody response (4; green).

The multiplex antibody assay was employed to analyze humoral immune responses (the multiplex panel included seven human coronaviruses) in the plasma of COVID-19 patients and non-COVID-19 respiratory disease controls (Figure 4). Antibodies detected by the multiplex panel that included SARS-CoV-2 S-RBD- and N, SARS-CoV S and N, MERS S-RBD, and S proteins of four common coronaviruses (229E, NL63, OC43, and HKU1), and they are shown in a heatmap (Figure 4).

Plasma samples from the majority of the COVID-19 patients were positive for antibodies against S-RBD and N of SARS-CoV-2 with some cross-reactivity to S and N proteins of SARS-CoV. This is likely due to the fact RBD in the S proteins of SARS-CoV-2 and SARS-CoV share 74% amino acid identity [24] and the N proteins of the two viruses contains share 90% amino acid identity [25]. The majority of the non-COVID-19 respiratory disease controls did not exhibit a significant background reactivity to SARS-CoV-2 S-RBD, SARS-CoV S, SARS-CoV N, and MER S-RBD. Antibodies against the four common coronaviruses were present in the majority of the COVID-19 patients and disease controls.

### 2.5. Antibodies against SARS-CoV-2 S-RBD Related to COVID-19 Severity

**Figure 5:**
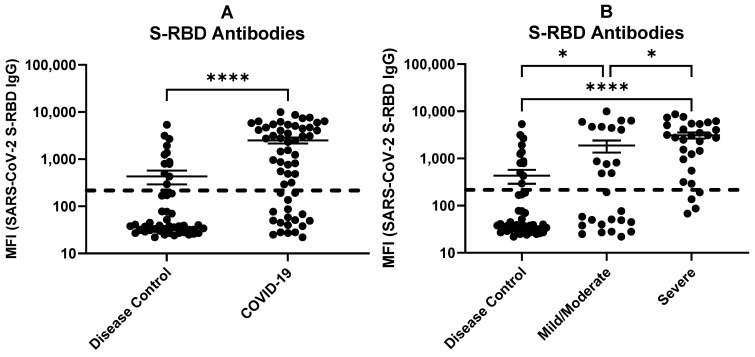
Antibodies against SARS-CoV-2 S-RBD in (**A**) COVID-19 patients (n = 56) and disease controls (n = 49). (**B**) COVID-19 patients stratified by disease severity (mild/moderate, n = 27; severe, n = 29) and non-COVID-19 respiratory disease controls (n = 49). Multiplex antibody assay was performed to detect antibodies against the following antigens: SARS-CoV-2 S-RBD and N, SARS-CoV S and N, MERS-CoV S-RBD, and S proteins of four common coronaviruses. Only antibodies against SARS-CoV-2 proteins are shown. MFI values (log10) with mean and SEM are shown. The dotted line indicates the assay cut-off values calculated using healthy controls (n = 101). *p*-values by Mann–Whitney and Kruskal–Wallis analyses between the groups are as follows: * *p* < 0.05, **** *p* < 0.0001.

Antibodies against SARS-CoV-2 S-RBD were significantly elevated in COVID-19 patients (mean MFI = 2483; n = 56) compared to non-COVID-19 respiratory disease controls (mean MFI = 432; n = 49) (*p*-value < 0.0001) (Figure 5A). These COVID-19 patients were stratified by disease severity (severe, n = 29; mild/moderate, n = 27) to assess antibody responses (Figure 5B). The levels of antibodies in plasma samples in severe COVID-19 patients were significantly higher (mean MFI = 3138) than in mild/moderate COVID-19 patients (mean MFI = 1877) (*p*-value < 0.05). Antibodies against SARS-CoV-2 S-RBD were not detected in 4 individuals in the severe group and 14 individuals in the mild/moderate group, while 12 individuals in the disease control group had anti-SARS-CoV-2 S-RBD antibodies probably because these individuals were at some point infected with SARS-CoV-2 with unnoticeable symptoms (see Section 3). High levels of humoral responses in severe COVID-19 indicate that such responses may be involved in driving inflammation. The lower level of humoral responses in mild/moderate COVID-19 patients might be associated with a milder inflammatory response. These results highlight the potential of humoral immune responses in driving COVID-19. As previously reported, anti-S-RBD antibody-virion complexes trigger inflammatory responses leading to severe COVID-19 [26].

## 3. Discussion

In this report, we demonstrate that mild/moderate and severe COVID-19 patients display differential profiles of cytokine/chemokine and eicosanoid storms in COVID-19 patients’ plasma. Profiles of these immune system modulators showed an inverse correlation with respect to disease severity, i.e., cytokines/chemokines were significantly elevated in severe COVID-19. In contrast, lipid mediators were significantly elevated in mild/moderate COVID-19, but their levels were almost down to control levels in severe patient samples.

SARS-CoV-2-infection-driven immune responses are a complex phenomenon involving innate immune responses, adaptive immunity, and immunomodulatory molecules secreted by multiple cell types of the immune system. A delicate balance attained by the immune responses appears to be necessary for the successful control of viral replication and host survival by the early and sufficient release of cytokines. Therefore, it is important to study not only the inflammatory and anti-inflammatory molecules driving the disease process but also the role of antiviral humoral immune responses [27,28].

Tuberculosis (TB) and COVID-19 are both lung diseases driven by inflammatory host responses. We have shown in active pulmonary TB that patients with weak humoral responses to *Mycobaterium tuberculosis* contain significantly higher levels of cytokines/chemokines, and patients with strong humoral responses contain significantly lower levels of cytokines/chemokines, suggesting TB patients who mount a strong Th1 response (cellular) do not have a concomitantly strong Th2-driven B-cell (humoral antibody) response [29,30]. Thus, differential cellular and humoral responses underly active TB, a chronic lung disease. We hypothesized a similar correlation would exist in COVID-19, another lung disease, but acute. Notably, data for COVID-19 patients in this study show that both cellular and humoral immune responses drive severe disease in unison.

Our results strongly suggest that in COVID-19, anti-S-RBD antibodies bound to the spike protein of the virus trigger stronger inflammatory responses, leading to severe disease. This conclusion is supported by a previous report that antibody-virion complexes stimulate the accumulation of pro-inflammatory macrophages and monocytes in the lungs as mediated by chemotaxis toward the cytokines [26]

In this study, in severe (when compared to mild/moderate) disease, several lipid mediators (TxB2, 11-HETE, 9-HODE, 13-HODE, 5-HETE, 12-HETE, 15-HETE, 14S-HDHA, 17S-HDHA, and 5-oxo ETE) were significantly reduced, whereas inflammatory cytokine/chemokines (IL-6, HGF, and CXCL-10) and humoral immune responses (anti-S-RBD antibodies) were elevated. Such a significant reduction in the levels of the above lipid mediators in severe versus mild/moderate disease might suggest that the lipid mediator release is significantly higher in moderate disease compared to severe disease. However, this observation could also imply that the lipid storm in response to viral infection is not physiologically sustained throughout the development of severe disease. We were particularly interested in seeing elevations in resolving lipids (resolvins, lipoxins, and moresins) during the course of the disease, but we have not seen that in this set of samples.

Taken together, the results in our study suggest that inflammatory processes leading to severe disease are driven by cytokine/chemokines and humoral immune responses together.

Although we set out to determine the changes in a number of lipid mediators (pro- and anti-inflammatory), we also had an untested minor hypothesis which states that changes in anti-inflammatory or pro-resolving lipid mediators together with the cytokine/chemokine levels might be better biomarkers of the severity of the disease. In this regard, it is noteworthy that, we did not see a clear indication of any of the pro-resolving lipid mediators in our analyses. Instead, we observed primarily pro-inflammatory lipid mediators that peaked in moderate patient samples. These findings are novel and have not been reported previously. In future studies, we plan to incorporate these findings into individual lipid/cytokine ratios and examine their utility in predicting the development of severe disease.

We previously reported a multiple reaction monitoring LC-MS/MS method to measure a panel of pro-inflammatory, anti-inflammatory, and pro-resolving lipid mediators [16]. This method quantitatively evaluates 39 of the bioactive signaling metabolites of LA, AA, DHA, and EPA in the COX and LOX pathways, together with pathway markers and stable end products, as described in the present study [16]. Of the lipid mediators that were quantified by this method, those with elevated concentrations in COVID-19 patient plasma have well-documented roles in immune response as well as diseases affecting the lung and heart (Figure 3) [2,17,20,21,23]. These lipid mediators include pro-inflammatory and anti-inflammatory/pro-resolving molecules. HETEs and HODEs can lead to vascular remodeling, cell proliferation, inflammation, vasoconstriction, angiogenesis, and fibrosis associated with pulmonary hypertension [17]. Leukotrienes, including LTB_4_, contribute to cell proliferation, resistance to apoptosis, and inflammation in the context of pulmonary arterial hypertension, and additionally, it contributes to neutrophil recruitment and vascular leakage [14,17]. TXB_2_, the inactive product of the biologically unstable TXA_2_, was increased in COVID-19 patients compared to disease controls (Figure 3). TXA_2_ chemical effects include increasing platelet aggregation and vasoconstriction while decreasing T cell activation [14]. 14S-HDHA and 17S-HDHA possess pro-resolving, pain-reducing, and tissue regenerative properties [19]. 17-HDHA in particular can limit neutrophil infiltration and regulate macrophages [19]. Our findings are also similar to other studies that have shown increased levels of eicosanoids in the lung and plasma of COVID-19 patients [20,21]. It is also interesting to note that blockage of eicosanoids utilizing tools such as apoA-I mimetic peptides modulates inflammatory bowel disease, pulmonary hypertension, and lung cancer in mice [16,18,31,32]. With regards to COVID-19 pathophysiology, blockage of eicosanoid signaling protects mice from severe disease, and apoA-I mimetics attenuate replication of the virus, apoptosis, inflammation, and oxidative stress in vitro [33,34]. In combination with these reports, targeting eicosanoids and their signaling pathways may be key to potential treatments and therapeutics for COVID-19.

Several studies have shown that the level of inflammatory cytokines is increased in COVID-19 [35,36,37,38]. The stimulation of an effective Th1 response is a characteristic of SARS-CoV-2 infection [39]. However, the cytokines storm in severe COVID-19 can induce a Th2 response against SARS-CoV-2, with poor COVID-19 prognosis [40]. The difference in the Th1/Th2 balance in SARS-CoV-2 infection has been linked to disease outcomes [41]. In the present study, all COVID-19 patients exhibited significantly higher levels of IL-6, CXCL-10, HGF, MIG, MCP-1, and G-CSF than the non-COVID-19 respiratory disease controls. Severe COVID-19 patients had elevated levels of cytokines, IL-6, CXCL-10, and HGF compared to mild/moderate. IL-6 has been reported to be the leading cause of inflammatory response in severe COVID-19 and emerged as an important regulator of Th1/Th2 differentiation, promoting the IL-4-dependent induction of Th2 differentiation and inhibiting Th1 differentiation [42,43]. The role of IL-6 in inflammation in insulin target tissues, the pathogenesis of systemic insulin resistance, and increasing vascular permeability contributing to tissue damage have been reported [44,45,46]. The role of HGF in inducing monocyte-macrophage activation, B-cell homing, and modulation of dendritic cell functions has been reported [47,48,49]. We and others have reported that CXCL-10, the most prominently elevated cytokine in COVID-19 patients in this study, is a useful inflammatory marker related to COVID-19 [13,50,51]. A study from Mexico reported elevated levels of MIG, MCP-1, and CXCL-10 in severe COVID-19 patients, consistent with our findings [52]. Previously, we described elevated levels of cytokines/chemokines such as IL-18, IFN-γ, CXCL-10, CXCL-9, G-CSF, IL-6, CXCL-1, VEGF, and PDGF-BB in plasma samples of tuberculosis (TB) patients with active pulmonary disease [29,30]. Even though the cytokine storm is observed in both *Mycobacterium tuberculosis* (*M*.*tb*) and SARS-CoV-2 infection, the outcomes associated with the cytokine storm are different in both diseases [53]. The cytokine storm in COVID-19 leads to acute respiratory distress syndrome (ARDS), whereas in TB it leads to chronic and slow lung destruction [53].

Interestingly, we observed higher levels of lipid mediators and lower levels of cytokines/chemokines in mild/moderate compared to severe COVID-19, and vice versa. This seems counterintuitive as it has been reported that the eicosanoid storm leads to the cytokine storm in COVID-19 infection [23]. However, some studies reported that lipid mediator profiles are dysregulated in critically ill COVID-19 patients, including decreased levels of pro-inflammatory eicosanoids [21]. It is also noteworthy that eicosanoids are short-lived autocrine and paracrine molecules [54]. Given that it takes days to go from infected to a severe state of illness, it may be possible that once the eicosanoids have initiated the inflammasome and stimulated cytokine release, both of which would lead to severe outcomes, the eicosanoid levels decrease. Unfortunately, no patients in this study were monitored over time. Such a longitudinal study would be important to determine how lipid levels change as the illness progresses from mild to severe stages.

Anti-SARS-CoV-2 antibodies were significantly elevated in severe COVID-19 patients compared to mild/moderate disease; this finding is in agreement with previous studies [55,56,57]. The underlying mechanism which establishes the relationship between anti-SARS-CoV-2 antibody levels and COVID-19 disease severity is not yet elucidated. Higher viral load in severe COVID-19 than in mild/moderate disease may lead to increased antibody levels. In our study, 52% of individuals in the mild/moderate COVID-19 group lacked antibodies, whereas 14% in the severe group lacked antibodies. Our data are consistent with previous studies showing that IgG antibodies are less likely to develop in persons with mild/moderate disease [58,59,60]. PCR tests can detect SARS-CoV-2 during the period of viral shedding but the duration of viral shedding is not well understood [61]. Importantly, RT-PCR may yield up to 29 percent false-negative results globally; 10 percent in the USA [62]. Among the non-COVID-19 respiratory disease controls, 24% of patients were antibody positive. The presence of anti-SARS-CoV-2 antibodies in some of the disease controls indicates that these patients might have previously been infected with the virus but may have remained asymptomatic. These samples were collected in 2021 when the pandemic was at its peak. These disease controls may not have been infected with the virus when the samples were collected but might have been previously infected. These samples were negative by SARS-CoV-2 RT-PCR, which suggests that these patients may not have had a viral infection at the time of sample collection.

To summarize, our findings indicate that SARS-CoV-2 infection can lead to robust activation pathways that regulate the resolution of the disease by generating pro-inflammatory cytokines and lipid mediators. Further investigation of these pathways is needed to support the development of novel anti-inflammatory treatments targeted to these pathways. A thorough understanding of the interplay of cytokines and lipid mediators is crucial for developing strategies for the management of SARS-CoV-2 infection.

## 4. Materials and Methods

### 4.1. Ethics Statement

Plasma from COVID-19 patients and disease controls were obtained under the protocols approved through the relevant Institutional Review Boards (IRBs) at the University of California, Davis Medical Center, and National Institute of Health (NIH), Pakistan (IRB 758309-1). Written informed consent was received from all participants before inclusion in the study and all the samples were de-identified before access.

### 4.2. COVID-19 Patient Plasma Samples

All COVID-19 patients were confirmed by RT-PCR testing for SARS-CoV-2. The date of illness onset, clinical classification, RT-PCR testing results during the hospitalization period, and personal demographic information were obtained from the clinical records. EDTA-treated blood samples were drawn, processed, and stored under a standardized protocol: blood was kept on ice immediately after collection, and plasma was separated by centrifugation at 1000× *g*, 10 min, (room temperature) within 2 h of collection and immediately frozen in aliquots at −80 °C until use. This blood plasma collection method is suitable for cytokines/chemokines but may not be ideal for lipid mediators [13,63,64].

Plasma samples (n = 56) were collected between May 2020 and November 2021 from the National Institutes of Health (NIH), Pakistan. COVID-19 patients were stratified by disease severity. Patients with mild or moderate symptoms who did not require admittance to intensive care were classified as mild to moderate COVID-19 (n =27), whereas patients who developed severe symptoms and required admittance to intensive care were classified as severe COVID-19 (n = 29). Basic demographic and clinical information of the SARS-CoV-2 patient from Pakistan included in the study can be found in Table S1. The median age of the severe patients was 60 years (IQR: 50–68 years); the median age of the mild/moderate group was 49 years (IQR: 34–63 years). None of the study subjects received COVID-19 vaccine at the time of sample collection.

### 4.3. Respiratory Disease Control Patient Samples (Other Than COVID-19)

Patients with other respiratory illnesses who were negative for SARS-CoV-2 PCR served as disease controls (n = 49) (median age: 43 years; IQR: 30–53 years) in this study. Basic demographic and clinical information of the disease controls is shown in Supplementary Table S1.

### 4.4. Healthy Control Group

These samples served as negative controls in this study; they were collected before the SARS-CoV-2 pandemic and cryopreserved at −80 °C. This group comprised pre-pandemic EDTA blood samples of healthy individuals (n = 54) of mixed sex (median age: 21 years; IQR: 20, 23) taken from Pakistan; these individuals had no history of pulmonary symptoms, and no known medical conditions (infection, cancer, or metabolic disease) [64]. This group consisted of random, young individuals to represent the general healthy population for comparison to COVID-19 patients.

### 4.5. Multiplex Antibody Assay

Multiplex assays were performed based on the xMAP platform (Luminex Corp, Austin, TX, USA) and median fluorescence intensity (MFI) data were collected as previously described [13]. Briefly, plasma samples were diluted 1:200 in 2% Prionex and incubated with HCoV antigen-coated beads for 1 h at room temperature in a 96-well plate. After incubation, the beads were washed twice by adding 100 μL of wash buffer (PBS-tween) per well and drained under vacuum using a vacuum manifold (Millipore Corporation, Bedford, MA, USA). To detect human IgG, phycoerythrin-conjugated anti-human IgG was used (Jackson Immuno Research, PA, USA) at a 1:500 dilution in PBS-tween and incubated at room temperature for 15 min. Following incubation, beads were washed twice with wash buffer, resuspended in 100 μL of wash buffer per well, and analyzed in the Magpix instrument.

We have previously published the relative quantitation of antibody responses to SARS-CoV-2 S-RBD based on a standard curve (MFI values plotted against serial dilutions of a standard plasma sample) and shown that the MFI values strongly correlated with antibody titers in longitudinal samples collected from COVID-19 patients [13]. We have also shown that pre-existing immunity to common coronaviruses does not confer cross-reactivity against SARS-CoV-2 infection [13]. Variations in the protein sequence between common HCoVs and SARS-CoV-2 RBDs may account for this lack of cross-reactivity [13,65].

### 4.6. Multiplex Cytokine/Chemokine Assay

Multiplex kits for measuring cytokines, chemokines, and growth factors (Cat#12007283), for use on the Luminex platform (Luminex Corp., Austin, TX, USA), were obtained from Bio-Rad, Hercules, CA. Assays were performed according to the manufacturer’s instructions. There were 48 immune molecules/analytes (cytokines/chemokines) in the assay kit that included: FGF basic, Eotaxin, G-CSF, GM-CSF, IFN-γ, IL-1β, IL-1ra, IL-1α, IL-2Rα, IL-3, IL-12 (p40), IL-16, IL-2, IL-4, IL-5, IL-6, IL-7, IL-8, IL-9, GRO-α, HGF, IFN-α2, LIF, MCP-3, IL-10, IL-12 (p70), IL-13, IL-15, IL-17A, CXCL-10 (IP-10), MCP-1 (MCAF), MIG, β-NGF, SCF, SCGF-β, SDF-1α, MIP-1α, MIP-1β, PDGF-BB, RANTES, TNF-α, VEGF, CTACK, MIF, TRAIL, IL-18, M-CSF, and TNF-β. The concentration (pg/mL) of each cytokine/chemokine in the multiplex panels was measured based on a 7-point standard curve using xPONENT 4.3 software (Luminex, TX, USA).

### 4.7. Lipidomics

Human patient plasma samples were used for measurements of oxidized lipids using liquid chromatography/tandem mass spectrometry analysis (LC-MS/MS). A detailed protocol to detect 39 distinct bioactive lipids, degradation products, and pathway markers has been described in the study by Meriwether et al. [16]. 18-hydroxyeicosapentaenoic acid (18-HEPE); 6-keto-prostaglandin F1α (6kPGF1α); resolvin E1 (RvE1); resolvin E1 deuterated (RvE1-d4); thromboxane B3 (TXB3); thromboxane B2 (TXB2); thromboxane B2 deuterated (TXB2-d4); prostaglandin E3 (PGE3); 20-hydroxy-leukotriene B4 (LTB4); prostaglandin F2α (PGF2α); prostaglandin E2 (PGE2); prostaglandin E2 deuterated (PGE2-d4); resolvin D3 (RvD3); resolvin D3 deuterated (RvD3-d5); lipoxin B4 (LXB4), prostaglandin D2 (PGD2); prostaglandin D2 deuterated (PGD2-d4); resolvin D2 (RVD2); resolvin D2 deuterated (RvD2-d5); leukotriene C4 (LTC4); leukotriene C4 deuterated (LTC4-d5); 15-keto-prostaglandin E2 (15keto-PGE2); leukotriene E4 (LTE4); lipoxin A4 (LxA4); lipoxin A4 deuterated (LxA4-d5); 15-epi-lipoxin A4 (15epi-LXA4); resolvin D1 (RvD1); resolvin D1 deuterated (RvD1-d5); 13, 14-dihydro-15-keto prostaglandin F2α (13,14-dihydro-15ketoPGF2α); 13, 14-dihydro-15-keto prostaglandin F2α deuterated (13,14-dihydro-15-keto-PGF2α-d4); 13, 14-dihydro-15-keto prostaglandin E2 (13,14-dihydro-15keto-PGE2); 13, 14-dihydro-15-keto prostaglandin E2 deuterated (13,14-dihydro-15-keto-PGE2-d4); resolvin D4 (RvD4); 13, 14-dihydro-15- keto prostaglandin D2 (13,14-dihydro-15keto-PGD2); prostaglandin J2 (PGJ2); Δ12-Prostaglandin J2 (Δ12-PGJ2); 7(S)-maresin (MaR1); 7(S)-maresin deuterated (MaR1-d5); 10(S),17(S)-protectin (PDx); resolvin D5 (RvD5); leukotriene B4 (LTB4); leukotriene B4 deuterated (LTB4-d4); 15-deoxy-Δ12,14-Prostaglandin J2 (15d-PGJ2); 15-deoxy-Δ12,14-Prostaglandin J2 deuterated (15d-PGJ2-d4); 13-hydroxyoctadecadienoic acid (13-HODE), 9-HODE, 13-HODE-d4; 15-hydroxyeicosatetraenoic acid (15-HETE) and 12-HETE, 5-HETE, 15-HETE-d8, 12-HETE-d8, 5-HETE-d8, and 11-HETE; 17S-hydroxydocosahexaenoic acid (17S-HDHA) and 14S-HDHA; 5-oxoeicosatetraenoic acid (5- oxoETE); and 5-oxoeicosatetraenoic acid deuterated (5-oxoETE-d7) were purchased from Cayman Chemicals (Ann Arbor, MI, USA) and used to create standard curves and internal standard mixtures.

To deactivate the virus, samples were treated by adding 450 uL of 100% methanol to a 50 uL sample to achieve a final concentration of 90% methanol. Samples were then incubated for 30 min at room temperature before freezing. A total of 100 μL of the sample was used from 500 uL of stock and combined with 150 µL of methanol, 1 µL of 20 mM butylated hydroxytoluene (BHT), and 50 µL of the internal standard mixture in methanol [16]. The samples were vortexed and left at −80 °C for 30 min before centrifuging them at 13,000 rpm for 10 min. The supernatant was then combined with 1.8 mL of acidified water (pH 3–4). The resulting sample was loaded onto a preconditioned 3cc Oasis hydrophilic-lipophilic-balanced (HLB) solid phase extraction (SPE) cartridge on a vacuum manifold (Waters). The SPE cartridge was equilibrated with 2 mL of methanol followed by 2 mL of water before the sample load. The sample was slowly loaded on the cartridge, and the cartridge was washed with 2 mL of 5% methanol in water. The lipids were subsequently eluted with 2 mL of methanol. The eluate was then evaporated to dryness under a stream of argon. A total of 100 μL of methanol was added to the dried extract, vortexed for 30 s, and the reconstituted extract was centrifuged at 13,000 rpm for 10 min to remove any precipitate that could clog the LC/MS/MS instrument [16]. The resulting supernatants were transferred to autosampler vials and processed for LC/MS/MS analysis. Quantification was performed using Analyst 1.6.3 and MultiQuant software 3.0.

### 4.8. Data Analysis

For the analysis of antibody data, cut-off values were calculated for each antigen-coated microbead set using data from healthy individuals (Cut-off = Mean MFI + (3 standard deviations)). The cut-off values were used to determine antibody-positive samples in the data sets [13].

For measurements of antibodies, cytokines/chemokines, and lipid mediators, graphs were generated and *p*-values were determined by a Mann–Whitney test when comparing disease controls versus all COVID-19 patients using GraphPad Prism. To compare differences between disease control, mild/moderate COVID-19, and severe COVID-19 groups, the data were tested with a one-way Kruskal–Wallis test using GraphPad Prism, and adjusted *p*-values were reported. The heatmap was generated using Prism after normalizing the data by log2(n + 1) for lipids and cytokines/chemokines, and log2 for all the antibody data.

## Data Availability

All data available in the supplementary files.

## Supplementary Materials

The following supporting information can be downloaded at: https://www.mdpi.com/article/10.3390/ijms241713054/s1.

## References

[1] Organization WHO (2023). WHO Coronavirus Disease (COVID-19) Dashboard with Vaccination Data|WHO Coronavirus (COVID-19) Dashboard With Vaccination Data. https://covid19.who.int/.

[2] Estenssoro E., Loudet C.I., Dubin A., Edul V.S.K., Plotnikow G., Andrian M., Sagardía J., Bezzi M., Mandich V., Groer C. (2022). Clinical characteristics, respiratory management, and determinants of oxygenation in COVID-19 ARDS: A prospective cohort study. J. Crit. Care.

[3] Alsafi R.T. (2022). Lessons from SARS-CoV, MERS-CoV, and SARS-CoV-2 Infections: What We Know So Far. Can. J. Infect. Dis. Med. Microbiol..

[4] Dinarello C.A. (2000). Proinflammatory cytokines. Chest.

[5] Wright T.M., Feghali C.A. (1997). Cytokines in acute and chronic inflammation. Front. Biosci..

[6] Kimura H., Yoshizumi M., Ishii H., Oishi K., Ryo A. (2013). Cytokine production and signaling pathways in respiratory virus infection. Front. Microbiol..

[7] Menachery V.D., Eisfeld A.J., Schäfer A., Josset L., Sims A.C., Proll S., Fan S., Li C., Neumann G., Tilton S.C. (2014). Pathogenic Influenza Viruses and Coronaviruses Utilize Similar and Contrasting Approaches to Control Interferon-Stimulated Gene Responses. mBio.

[8] Soy M., Keser G., Atagündüz P., Tabak F., Atagündüz I., Kayhan S. (2020). Cytokine storm in COVID-19: Pathogenesis and overview of anti-inflammatory agents used in treatment. Clin. Rheumatol..

[9] Min C.-K., Cheon S., Ha N.-Y., Sohn K.M., Kim Y., Aigerim A., Shin H.M., Choi J.Y., Inn K.S., Kim J.H. (2016). Comparative and kinetic analysis of viral shedding and immunological responses in MERS patients representing a broad spectrum of disease severity. Sci. Rep..

[10] Giamarellos-Bourboulis E.J., Netea M.G., Rovina N., Akinosoglou K., Antoniadou A., Antonakos N., Damoraki G., Gkavogianni T., Adami M.E., Katsaounou P. (2020). Complex Immune Dysregulation in COVID-19 Patients with Severe Respiratory Failure. Cell Host Microbe.

[11] Lucas C., Wong P., Klein J., Castro T.B.R., Silva J., Sundaram M., Ellingson M.K., Mao T., Oh J.E., Israelow B. (2020). Longitudinal analyses reveal immunological misfiring in severe COVID-19. Nature.

[12] Mehta P., McAuley D.F., Brown M., Sanchez E., Tattersall R.S., Manson J.J. (2020). COVID-19: Consider cytokine storm syndromes and immunosuppression. Lancet.

[13] Ravindran R., McReynolds C., Yang J., Hammock B.D., Ikram A., Ali A., Bashir A., Zohra T., Chang W.L.W., Hartigan-O’Connor D.J. (2021). Immune response dynamics in COVID-19 patients to SARS-CoV-2 and other human coronaviruses. PLoS ONE.

[14] Dennis E.A., Norris P.C. (2015). Eicosanoid storm in infection and inflammation. Nat. Rev. Immunol..

[15] Litwack G. (2022). Eicosanoids. Hormones.

[16] Meriwether D., Sulaiman D., Volpe C., Dorfman A., Grijalva V., Dorreh N., Solorzano-Vargas R.S., Wang J., O’Connor E., Papesh J. (2019). Apolipoprotein A-I mimetics mitigate intestinal inflammation in COX2-dependent inflammatory bowel disease model. J. Clin. Investig..

[17] Sharma S., Ruffenach G., Umar S., Motayagheni N., Reddy S.T., Eghbali M. (2016). Role of Oxidized Lipids in Pulmonary Arterial Hypertension. Pulm. Circ..

[18] Ruffenach G., O’connor E., Vaillancourt M., Hong J., Cao N., Sarji S., Moazeni S., Papesh J., Grijalva V., Cunningham C.M. (2020). Oral 15-Hydroxyeicosatetraenoic Acid Induces Pulmonary Hypertension in Mice by Triggering T Cell–Dependent Endothelial Cell Apoptosis. Hypertension.

[19] Serhan C.N. (2014). Pro-resolving lipid mediators are leads for resolution physiology. Nature.

[20] Archambault A.S., Zaid Y., Rakotoarivelo V., Turcotte C., Doré É., Dubuc I., Martin C., Flamand O., Amar Y., Cheikh A. (2021). High levels of eicosanoids and docosanoids in the lungs of intubated COVID-19 patients. FASEB J..

[21] Palmas F., Clarke J., Colas R.A., Gomez E.A., Keogh A., Boylan M., McEvoy N., McElvaney O.J., McElvaney O., Alalqam R. (2021). Dysregulated plasma lipid mediator profiles in critically ill COVID-19 patients. PLoS ONE.

[22] Turnbull J., Jha R.R., Ortori C.A., Lunt E., Tighe P.J., Irving W.L., Gohir S.A., Kim D.H., Valdes A.M., Tarr A.W. (2022). Serum Levels of Proinflammatory Lipid Mediators and Specialized Proresolving Molecules Are Increased in Patients With Severe Acute Respiratory Syndrome Coronavirus 2 and Correlate with Markers of the Adaptive Immune Response. J. Infect. Dis..

[23] Hammock B.D., Wang W., Gilligan M.M., Panigrahy D. (2020). Eicosanoids: The Overlooked Storm in Coronavirus Disease 2019 (COVID-19)?. Am. J. Pathol..

[24] Jaimes J.A., André N.M., Chappie J.S., Millet J.K., Whittaker G.R. (2020). Phylogenetic Analysis and Structural Modeling of SARS-CoV-2 Spike Protein Reveals an Evolutionary Distinct and Proteolytically Sensitive Activation Loop. J. Mol. Biol..

[25] Dutta N.K., Mazumdar K., Gordy J.T. (2020). The Nucleocapsid Protein of SARS-CoV-2: A Target for Vaccine Development. J. Virol..

[26] Montazersaheb S., Khatibi S.M.H., Hejazi M.S., Tarhriz V., Farjami A., Sorbeni F.G., Farahzadi R., Ghasemnejad T. (2022). COVID-19 infection: An overview on cytokine storm and related interventions. Virol. J..

[27] Shivshankar P., Karmouty-Quintana H., Mills T., Doursout M.F., Wang Y., Czopik A.K., Evans S.E., Eltzschig H.K., Yuan X. (2022). SARS-CoV-2 Infection: Host Response, Immunity, and Therapeutic Targets. Inflammation.

[28] Park A., Iwasaki A. (2020). Type I and Type III Interferons—Induction, Signaling, Evasion, and Application to Combat COVID-19. Cell Host Microbe.

[29] Ravindran R., Krishnan V.V., Khanum A., Luciw P.A., Khan I.H. (2013). Exploratory study on plasma immunomodulator and antibody profiles in tuberculosis patients. Clin. Vaccine Immunol..

[30] Chavez K., Ravindran R., Dehnad A., Khan I.H. (2016). Gender biased immune-biomarkers in active tuberculosis and correlation of their profiles to efficacy of therapy. Tuberculosis.

[31] Sharma S., Umar S., Potus F., Iorga A., Wong G., Meriwether D., Breuils-Bonnet S., Mai D., Navab K., Ross D. (2014). Apolipoprotein A-I Mimetic Peptide 4F Rescues Pulmonary Hypertension by Inducing MicroRNA-193-3p. Circulation.

[32] Chattopadhyay A., Yang X., Mukherjee P., Sulaiman D., Fogelman H.R., Grijalva V., Dubinett S., Wasler T.C., Manash K., Salehi-Rad P.R. (2018). Treating the Intestine with Oral ApoA-I Mimetic Tg6F Reduces Tumor Burden in Mouse Models of Metastatic Lung Cancer. Sci. Rep..

[33] Wong L.-Y.R., Zheng J., Wilhelmsen K., Li K., Ortiz M.E., Schnicker N.J., Thurman A., Pezzulo A.A., Szachowicz P.J., Li P. (2022). Eicosanoid signalling blockade protects middle-aged mice from severe COVID-19. Nature.

[34] Kelesidis T., Madhav S., Petcherski A., Cristelle H., O’Connor E., Hultgren N.W., Ritou E., Williams D.S., Shirihai O.S., Reddy S.T. (2021). The ApoA-I mimetic peptide 4F attenuates in vitro replication of SARS-CoV-2, associated apoptosis, oxidative stress and inflammation in epithelial cells. Virulence.

[35] Huang C., Wang Y., Li X., Ren L., Zhao J., Hu Y., Zhang L., Fan G., Xu J., Gu X. (2020). Clinical features of patients infected with 2019 novel coronavirus in Wuhan, China. Lancet.

[36] Chang S., Minn D., Kim S.-W., Kim Y. (2021). Inflammatory Markers and Cytokines in Moderate and Critical Cases of COVID-19. Clin. Lab..

[37] Kunnumakkara A.B., Rana V., Parama D., Banik K., Girisa S., Henamayee S., Thakur K.K., Dutta U., Garodia P., Gupta S.C. (2021). COVID-19, cytokines, inflammation, and spices: How are they related?. Life Sci..

[38] Perreau M., Suffiotti M., Marques-Vidal P., Wiedemann A., Levy Y., Laouénan C., Ghosn J., Fenwick C., Comte D., Roger T. (2021). The cytokines HGF and CXCL13 predict the severity and the mortality in COVID-19 patients. Nat. Commun..

[39] Shrotri M., van Schalkwyk M.C.I., Post N., Eddy D., Huntley C., Leeman D., Rigby S., Williams S.V., Bermingham W.H., Kellam P. (2021). T cell response to SARS-CoV-2 infection in humans: A systematic review. PLoS ONE.

[40] McGregor R., Chauss D., Freiwald T., Yan B., Wang L., Nova-Lamperti E., Zhang Z., Teague H., West E.E., Bibby J. (2020). An autocrine Vitamin D-driven Th1 shutdown program can be exploited for COVID-19. bioRxiv.

[41] Aleebrahim-Dehkordi E., Molavi B., Mokhtari M., Deravi N., Fathi M., Fazel T., Mohebalizadeh M., Koochaki P., Shobeiri P. (2022). Hasanpour-Dehkordi AT helper type (Th1/Th2) responses to SARS-CoV-2 and influenza A (H1N1) virus: From cytokines produced to immune responses. Transpl. Immunol..

[42] Park M.D. (2020). Macrophages: A Trojan horse in COVID-19?. Nat. Rev. Immunol..

[43] Roncati L., Ligabue G., Fabbiani L., Malagoli C., Gallo G., Lusenti B., Nasillo V., Manenti A., Maiorana A. (2020). Type 3 hypersensitivity in COVID-19 vasculitis. Clin. Immunol..

[44] Rocha V.Z., Folco E.J. (2011). Inflammatory Concepts of Obesity. Int. J. Inflamm..

[45] El-Kadre L.J., Tinoco A.C. (2013). Interleukin-6 and obesity: The crosstalk between intestine, pancreas and liver. Curr. Opin. Clin. Nutr. Metab. Care..

[46] Marin V., Montero-Julian F.A., Grès S., Boulay V., Bongrand P., Farnarier C., Kaplanski G. (2001). The IL-6-soluble IL-6Ralpha autocrine loop of endothelial activation as an intermediate between acute and chronic inflammation: An experimental model involving thrombin. J. Immunol..

[47] Galimi F., Cottone E., Vigna E., Arena N., Boccaccio C., Giordano S., Naldini L., Comoglio P.M. (2001). Hepatocyte Growth Factor Is a Regulator of Monocyte-Macrophage Function. J. Immunol..

[48] van der Voort R., Taher T.E., Keehnen R.M., Smit L., Groenink M., Pals S.T. (1997). Paracrine Regulation of Germinal Center B Cell Adhesion through the c-Met–Hepatocyte Growth Factor/Scatter Factor Pathway. J. Exp. Med..

[49] Singhal E., Kumar P., Sen P. (2011). A novel role for Bruton’s tyrosine kinase in hepatocyte growth factor-mediated immunoregulation of dendritic cells. J. Biol. Chem..

[50] Zhang N., Zhao Y.-D., Wang X.-M. (2020). CXCL10 an important chemokine associated with cytokine storm in COVID-19 infected patients. Eur. Rev. Med. Pharmacol. Sci..

[51] Blot M., Jacquier M., Glele L.-S.A., Beltramo G., Nguyen M., Bonniaud P., Prin S., Andreu P., Bouhemad B., Bour J.-B. (2020). CXCL10 could drive longer duration of mechanical ventilation during COVID-19 ARDS. Crit. Care.

[52] Ochoa-Ramirez L.A., Ramos-Payan R., Jimenez-Gastelum G.R., Rodriguez-Millan J., Aguilar-Medina M., Rios-Tostado J.J., Ayala-Ham A., Bermudez M., Osuna-Ramos J.F., Olimon-Andalon V. (2022). The Chemokine MIG is Associated with an Increased Risk of COVID-19 Mortality in Mexican Patients. Iran. J. Immunol..

[53] Tapela K., Ochieng’ Olwal C., Quaye O. (2020). Parallels in the pathogenesis of SARS-CoV-2 and M. tuberculosis: A synergistic or antagonistic alliance?. Future Microbiol..

[54] Biringer R.G. (2020). The enzymology of human eicosanoid pathways: The lipoxygenase branches. Mol. Biol. Rep..

[55] Yan X., Chen G., Jin Z., Zhang Z., Zhang B., He J., Yin S., Huang J., Fan M., Li Z. (2022). Anti-SARS-CoV-2 IgG levels in relation to disease severity of COVID-19. J. Med. Virol..

[56] Garcia-Beltran W.F., Lam E.C., Astudillo M.G., Yang D., Miller T.E., Feldman J., Hauser B.M., Caradonna T.M., Clayton K.L., Nitido A.D. (2021). COVID-19-neutralizing antibodies predict disease severity and survival. Cell.

[57] Long Q.-X., Liu B.-Z., Deng H.-J., Wu G.-C., Deng K., Chen Y.-K., Liao P., Qiu J.F., Lin Y., Cai X.F. (2020). Antibody responses to SARS-CoV-2 in patients with COVID-19. Nat. Med..

[58] Petersen L.R., Sami S., Vuong N., Pathela P., Weiss D., Morgenthau B.M., Henseler R.A., Daskalakis D.C., Atas J., Patel A. (2020). Lack of Antibodies to Severe Acute Respiratory Syndrome Coronavirus 2 (SARS-CoV-2) in a Large Cohort of Previously Infected Persons. Clin. Infect. Dis..

[59] Wellinghausen N., Voss M., Ivanova R., Deininger S. (2020). Evaluation of the SARS-CoV-2-IgG response in outpatients by five commercial immunoassays. GMS Infect. Dis..

[60] Robbiani D.F., Gaebler C., Muecksch F., Lorenzi J.C.C., Wang Z., Cho A., Agudelo M., Barnes C.O., Gazumyan A., Finkin S. (2020). Convergent antibody responses to SARS-CoV-2 in convalescent individuals. Nature.

[61] Vetter P., Eckerle I., Kaiser L. (2020). COVID-19: A puzzle with many missing pieces. BMJ.

[62] Arevalo-Rodriguez I., Buitrago-Garcia D., Simancas-Racines D., Zambrano-Achig P., Del Campo R., Ciapponi A., Sued O., Martinez-García L., Rutjes A.W., Low N. (2020). False-negative results of initial RT-PCR assays for COVID-19: A systematic review. PLoS ONE.

[63] Khan I.H., Ravindran R., Krishnan V.V., Awan I.N., Rizvi S.K., Saqib M.A., Shahzad M.I., Tahseen S., Ireton G., Goulding C.W. (2011). Plasma antibody profiles as diagnostic biomarkers for tuberculosis. Clin. Vaccine Immunol..

[64] Khaliq A., Ravindran R., Hussainy S.F., Krishnan V.V., Ambreen A., Yusuf N.W., Irum S., Rashid A., Jamil M., Zaffar F. (2017). Field evaluation of a blood based test for active tuberculosis in endemic settings. PLoS ONE.

[65] Benson D.A., Cavanaugh M., Clark K., Karsch-Mizrachi I., Lipman D.J., Ostell J., Sayers E.W. (2013). GenBank. Nucleic Acids Res..

